# Adolescent Neurodevelopmental Variance Across Social Strata

**DOI:** 10.1001/jamanetworkopen.2024.10441

**Published:** 2024-05-08

**Authors:** Katherine L. Bottenhorn, Carlos Cardenas-Iniguez, Jared N. Schachner, Michael A. Rosario, Kathryn L. Mills, Angela R. Laird, Megan M. Herting

**Affiliations:** 1 Department of Population and Public Health Sciences, University of Southern California, Los Angeles; 2 Department of Psychology, Florida International University, Miami; 3 Price School of Public Policy, University of Southern California, Los Angeles; 4 Department of Psychology, University of Oregon, Eugene; 5 Department of Physics, Florida International University, Miami

## Abstract

This cohort study explores variability in neurodevelopment across sociodemographic factors among youths.

## Introduction

Robust studies of human brain development require large, diverse pediatric samples^1^ to inform personalized medicine in pediatrics. Historically, neuroimaging research has overrepresented non-Hispanic White (hereinafter, White), wealthier populations, omitting both participants and researchers from marginalized racial, ethnic, and socioeconomic groups.^2^ Diversity and inclusion in pediatric research populations is vital, as is careful consideration of how social determinants of health contribute to downstream individual differences in neurodevelopmental trajectories. Despite progress describing normative (ie, average) adolescent neurodevelopment, individual variability remains poorly characterized. We investigated individual variability across sociodemographic factors in key facets of white and gray matter development, estimated from annual rates of change across early adolescence in the nationwide Adolescent Brain Cognitive Development (ABCD) Study.

## Methods

This cohort study used neuroimaging and demographic data from ABCD Study release 4.0, collected between 2016 and 2020 from 4131 to 7115 children (at ages 9-10 and 11-13 years) (eTable in Supplement 1). Brain change variance was compared across caregiver-reported sociodemographic factors, including child race and ethnicity, household income, and caregiver education.^3^ Caregivers and youths gave written consent and assent in accordance with institutional review board policy at each site. We calculated variance in annualized percent changes in cortical thickness, functional (blood oxygenation level–dependent) fluctuations, and white matter fractional anisotropy (FA) over 2 years (eMethods in Supplement 1). The Fligner-Killeen test was used to assess unequal variance in change scores across sociodemographic factors, with heteroscedasticity at α < .01 (family-wise error-rate corrected).^4^ The study followed the STROBE reporting guideline. Data analysis was performed from December 2021 to September 2022 using SciPy, version 1.9.1, in Python, version 3.8.9 (Python Software Foundation).

## Results

**Figure 1.**
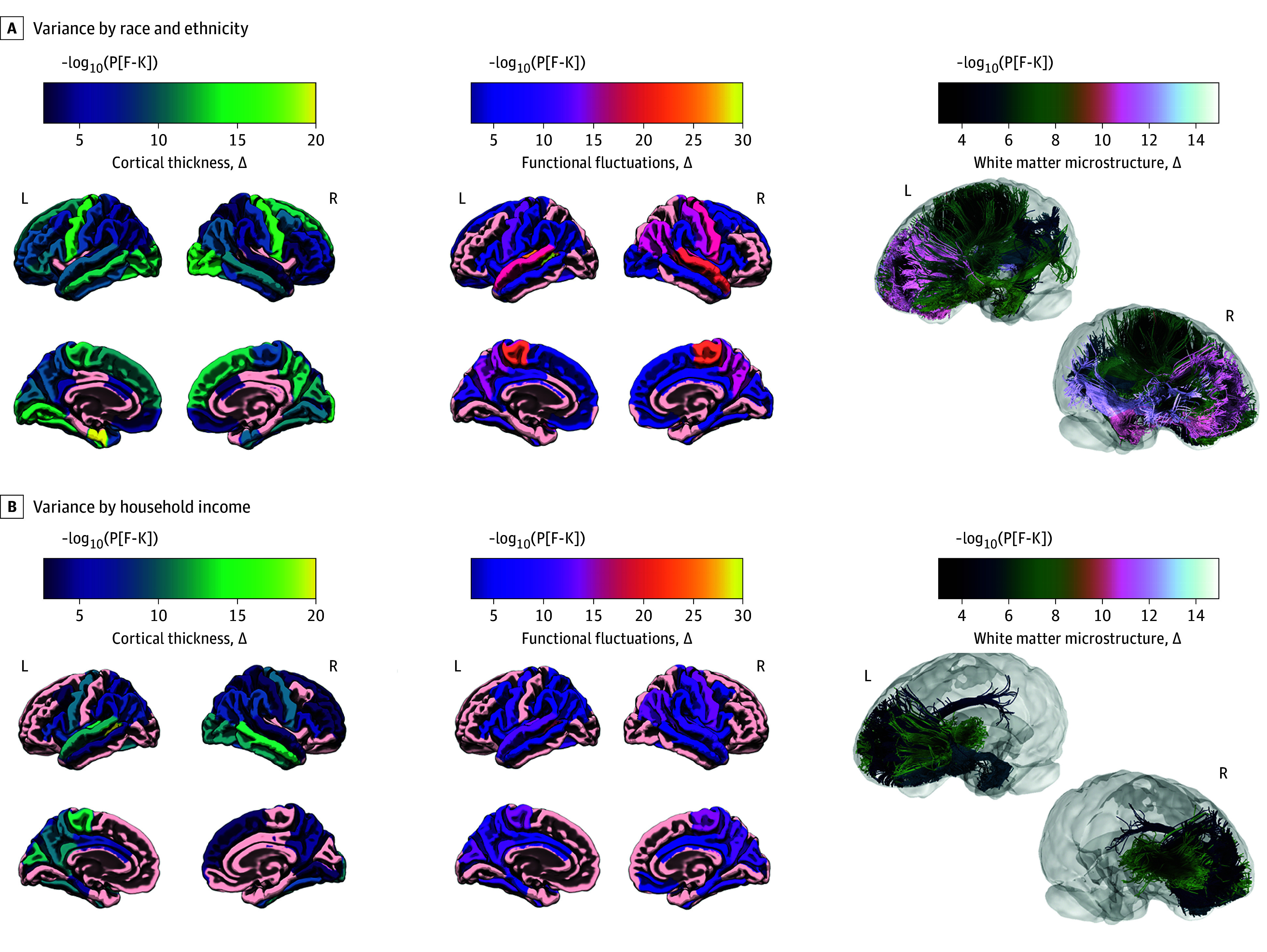
Significant Differences in Variance in Annual Rates of Structural and Functional Brain Development Along Sociodemographic Strata A and B, Heterogeneous neurodevelopmental variance across sociodemographic factors, including race and ethnicity (A) and household income (B), for cortical thickness (left), functional fluctuations (middle), and white matter microstructure (right). Cortical thickness is thought to reflect dendritic arborization and pruning. Functional fluctuations (middle) quantify the temporal variance of the blood oxygen level–dependent signal from functional MRI and are thought to reflect neural flexibility. White matter microstructure (right) refers to fractional anisotropy, a measure of white matter integrity that continues to mature across childhood and adolescence. Color coding indicates the negative logarithm of the *F* − *K* statistic *P* value, representing the degree of inhomogeneity of variance across strata, per sociodemographic variable. In the left and middle columns (ie, cortical thickness and functional fluctuations), 4 views of the brain’s surface are presented, including the left lateral (top left), right lateral (top right), left medial (bottom left), and right medial (bottom right). Because the right column presents white matter tracts inside the brain (ie, not on the cortical surface), only 2 views are presented, providing multiple angles from which to observe significantly heteroscedastic tracts. L indicates left; R, right.

This study included 7115 youths (3300 [46%] girls and 3815 [54%] boys; mean [SD] age, 9.9 [0.62] years at baseline data collection). Youths were Hispanic (1383 [19%]), non-Hispanic Black (924 [13%]), White (3938 [55%]), or other race or ethnicity (870 [12%]). We identified heteroscedasticity of annualized brain change across social strata from youths aged 8.9 to 13.0 years. Significant differences in variance were observed between racial and ethnic groups in rates of change in cortical thickness (81% of brain regions), functional fluctuations (63%), and white matter tract FA (63% of tracts) (Figure 1A). Household income (Figure 1B) and caregiver educational attainment also exhibited significant albeit less widespread heterogeneous variance, with heteroscedasticity of changes in cortical thickness (65% and 46%), white matter FA (49% and 23%), and functional fluctuations (55% and 58%), respectively. Differences in brain change variance were neuroanatomically similar across sociodemographic factors.

**Figure 2.**
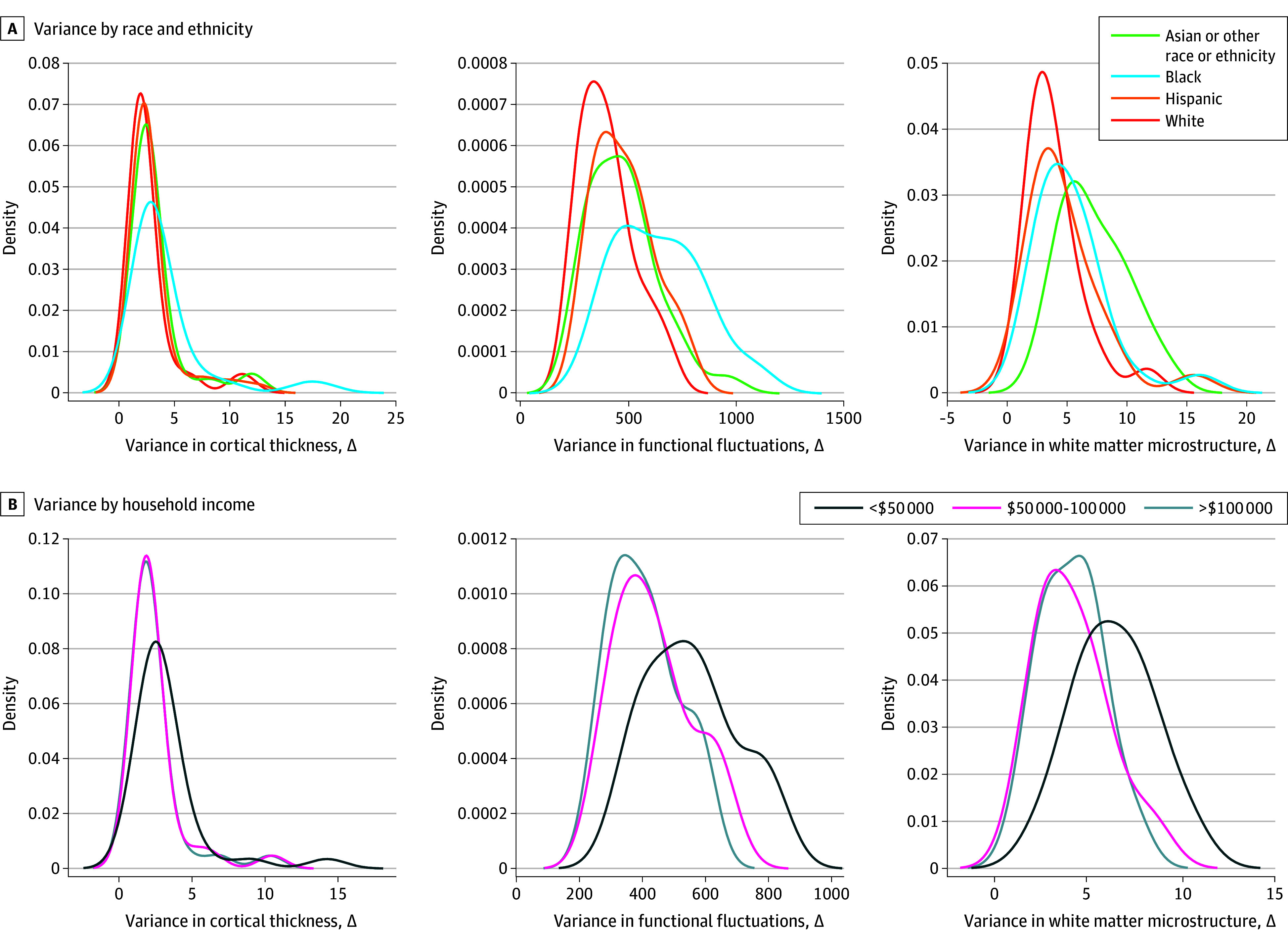
Variance in Developmental Changes Within Cortical Regions and White Matter Tracts Along Sociodemographic Strata A and B, Distribution of variance (x-axis) in annual rates of change, per brain region, in cortical thickness (left), functional fluctuations (middle), and white matter microstructure (ie, fractional anisotropy) (right). Distributions are color coded by strata across sociodemographic variables, including race and ethnicity (A) and household income (B). Caregiver education is not pictured. Statistically significant differences in variance (*P* < .01) were observed in (1) cortical thickness changes (top left) between non-Hispanic Black (hereinafter, Black) youths and both their Hispanic and non-Hispanic White (hereinafter, White) peers, (2) in functional fluctuations changes (top middle) between Black youths and their peers of each other race or ethnicity, and (3) in white matter microstructure changes (top right) between non-Hispanic Asian youths or youths of other race or ethnicity and White youths. Statistically significant differences were also observed (1) in cortical thickness (bottom left) and functional variance (bottom middle) changes between youths in households making less than $50 000 annually and households with incomes greater than $50 000 and (2) in white matter microstructure changes (bottom right) in youths in households making less than $50 000 annually and those in households with incomes greater than $100 000.

Across sociodemographic factors, the greatest variance in annual rates of brain development was observed between structurally disadvantaged individuals (eg, racial and ethnic minority children from low-income households with fewer years of caregiver education). The least variance in annual percent change was seen between structurally advantaged individuals (eg, White children from high-income households with greater caregiver education) (Figure 2).

## Discussion

We observed substantially different magnitudes of variance in annual rates of neurodevelopmental change across sociodemographic groups during early adolescence, which were greater than previously reported differences in variance regarding age, sex, and puberty in this same pediatric cohort.^5^ These findings suggest greater interindividual differences in neurodevelopment within marginalized and structurally disadvantaged groups compared with their more advantaged peers, potentially extending the previously identified variance-reducing effect of income^6^ to brain development.

Overlooking differences in neurodevelopmental variance across social and economic backgrounds may obscure determinants of brain health. Thus, population-level developmental neuroscience must clarify how individuals differ on key outcomes, both between and within socially stratified groups, especially in this dynamic and variable phase of early adolescence. Although this cohort study includes thousands of participants from across the US, it is limited by relying on coarse delineations of sociodemographic strata, with fewer participants from lower-income and racial and ethnic minority backgrounds compared with the general population. Future investigations are needed to develop and test theoretical models that elucidate how structural forces unfolding across generations shape individual children’s patterns of brain maturation.

## Notes

Supplement 1.
eMethods.
**eTable.** Demographic Composition of the Entire ABCD Study and the Samples Used Here
eReferences

Supplement 2.
Data Sharing Statement
