# Supplementary material for: Adolescent Neurodevelopmental Variance Across Social Strata
Source: JAMA Netw Open. 2024 May 8;7(5):e2410441. doi: 10.1001/jamanetworkopen.2024.10441 (PMC11079691; doi:10.1001/jamanetworkopen.2024.10441)
Supplement: Supplement 1. — eMethods. eTable. Demographic Composition of the Entire ABCD Study and the Samples Used Here eReferences [file jamanetwopen-e2410441-s001.pdf]

Supplementary Online Content

Bottenhorn KL, Cardenas-Iniguez C, Schachner JN, et al. Adolescent neurodevelopmental variance across social strata. *JAMA Netw Open*. 2024;7(5):e2410441. doi:10.1001/jamanetworkopen.2024.10441

eMethods

eTable. Demographic Composition of the Entire ABCD Study and the Samples Used Here

eReferences

This supplementary material has been provided by the authors to give readers additional information about their work.

© 2024 Bottenhorn KL et al. *JAMA Network Open.*

eMethods

Participants

Longitudinal data were collected as a part of the ongoing Adolescent Brain and Cognitive Development (ABCD) Study and included in the annual 4.0 data release (http://dx.doi.org/10.15154/1523041). The ABCD Study enrolled 11,880 children 9 to 10 years of age (mean age = 9.49; 48% female) in a 10-year longitudinal study. Participants were recruited at 21 study sites across the United States from elementary schools (private, public, and charter schools) in a sampling design that aimed to represent the nationwide sociodemographic diversity.[1] All experimental and consent procedures were approved by the institutional review board and human research protections programs at the University of California San Diego and each study site. ABCD Study inclusion criteria included age at enrollment (9.0 to 10.99 years); English fluency; lack of MRI contraindications; no history of traumatic brain injury or major neurological disorder; absence of any non-correctable sensory and/or motor impairments that would preclude the youth's participation in study procedures; current intoxication at appointment; diagnosis of any DSM-I psychotic, autism spectrum, or substance use disorder; an intellectual disability reported by their caregiver; premature birth, very low birth weight, or perinatal complications; and caregiver knowledge at baseline of an impending move to an area beyond reasonable traveling distance to an ABCD Study site. Each participant provided written assent to participate in the study and their legal guardian provided written agreement to participate. For more information, see Garavan et al.[1] and Volkow et al.[2] Here, we use a subset of data from the ABCD Study, including magnetic resonance imaging (MRI), in addition to measures of participants' socio-demographics. Data include assessments from two time points: baseline enrollment and year 2 follow-up. Measures of participants' demographics were all taken from the baseline time point.

Participants were excluded from these analyses if they did not have imaging data collected at the 2-year follow-up visit. From this subset, data were further excluded based on image quality. Structural and diffusion-weighted data were included if a participant's T1- and diffusion-weighted images, respectively, met the ABCD-recommended criteria for inclusion. For T1-weighted images, quality assessments were based on motion, intensity inhomogeneity, white matter and pial surface estimation by Freesurfer, and susceptibility artifacts. Exclusion was recommended if an image exhibited severe artifact in any of those categories. For diffusion-weighted images, quality assessments were based on residual B_0 distortion after processing, coregistration to the participant's T1, image quality, and segmentation quality, with exclusion recommended if an image exhibited severe artifact in any of those categories. For resting-state scans, quality assessments were based on the number of frames remaining after high-motion (i.e., framewise displacement > 0.3 mm) frames were censored and periods with fewer than 5 contiguous frames were excluded, coregistration to T1, B_0 distortion maps, Freesurfer tissue type segmentation quality, and presence of runs with fewer than 100 usable timepoints (after censoring and exclusion). We additionally excluded any resting-state fMRI data from participants with fewer than 750 usable (i.e., low-motion) frames, or 10 low-motion minutes, across all runs, based on estimations of scan lengths necessary for reliable resting-state functional connectivity estimates [3,4]. The final sample characteristics for the current study are described in eTable 1. Because each structural, diffusion-weighted, and resting-state functional data are included based on their image quality, there are different numbers of subjects included for analyses with different imaging modalities.

Demographic Data

All demographic data was reported by each participant's parent or legal guardian (hereafter, "caregiver") using the PhenX Toolkit.[5] From the shared 4.0 data release, variables were re-binned to ensure roughly equal-sized groupings for robust assessment of homogeneity of variance. Participants' race and ethnicity were reported by their caregiver as the race and ethnicity they considered their child to be, which was then grouped into five

© 2024 Bottenhorn KL et al. *JAMA Network Open.*

categories for consistency with other data sources and socioeconomic constructs: Non-Hispanic White, Non-Hispanic Black, Hispanic, and Other (including Non-Hispanic Asian, American Indian, Native Hawaiian, Samoan, Chamorro, Other Pacific Islander, belonging to more than one race (e.g., multiracial) and other racial and ethnic identities). Household income was reported by the caregiver as the total annual income, from all sources in the past calendar year, which we then re-binned in roughly equal-sized increments. Educational attainment was reported by the caregiver about their or their partner's own highest level of education, which we then re-binned based on postsecondary and higher education, in addition to degree attainment.

Neuroimaging Data

The ABCD Study's imaging protocol includes structural, diffusion, and both task-based and resting-state functional MRI collected every two years, as described by Casey et al.[6] The ABCD consortium has reported image processing and analysis methods in detail [7]. Important for multi-site studies, ABCD MRI methods and assessments have been optimized and harmonized across the 21 sites for 3 Tesla scanners (Siemens Prisma, General Electric 750, Philips).[6,7] Here, we assessed cortical thickness from structural T1-weighted MRI scans, functional fluctuations from blood-oxygenation-level-dependent (BOLD) functional MRI scans, and fractional anisotropy from diffusion-weighted MRI scans.

Analyses

An analysis plan was registered for this project with the Open Science Framework (OSF) and can be found here. Prior to data analysis, missingness was assessed across variables and for the purposes of assessing intra- and inter-individual variability only complete cases were used, per variable.

To assess inter-individual differences in brain changes, distributions of intra-individual differences were, first, compared across levels of the following sociodemographic variables using the Fligner-Killeen test for homogeneity of variances,[8] including combined household income; caregiver higher education, as well as participant's race and ethnicity as reported by the caregiver. Significance was assessed at $\alpha_{adjusted} < 0.01$, corrected for the number of effective comparisons across APΔ measures [9,10].

Then, post hoc rank-sum tests were performed for measures of brain change across significantly heteroscedastic demographic variables, to determine which levels of each variable demonstrate relatively more or less variance than others.

All code used to perform these analyses and generate the associated figures was written in Python 3.7 and is available at github.com/62442katieb/deltaABCD_variability. The following packages were used to run the reported analyses and create the figures included in this report: matplotlib (v.3.5.1), missingno (v.0.5.0), nibabel (v.3.2.1), nilearn (v.0.8.1), numpy (v.1.20.3), pandas (v.1.3.5), scipy (v.1.9.1), seaborn (v.0.11.2).

© 2024 Bottenhorn KL et al. *JAMA Network Open.*

eTable. Demographic Composition of the Entire ABCD Study and the Samples Used Here

	Total ABCD Study Sample	MRI 2-Year Follow-Up	sMRI Pass QC	dMRI Pass QC	rs-fMRI Pass QC
Total N (%)	11801	7457 (63%)	7115 (60%)	6250 (53%)	4131 (35%)
Age at baseline (months)	118.97±7.50	118.94 ± 7.44	118.82 ± 7.45	118.90 ± 7.46	119.46 ± 7.55
Sex (F)	5636 (48%)	3437 (46%)	3300 (46%)	2922 (47%)	2072 (50%)
Race & Ethnicity					
Other	1493	918	870	733	482
Hispanic	2402	1450	1383	1195	765
Non-Hispanic Black	1755	981	924	787	420
Non-Hispanic White	6149	4108	3938	3535	2464
Household Income					
>$100k	4543	2876	2760	2485	1719
$50 to $100k	3056	2082	1992	1757	1195
<$50k	3194	1922	1817	1533	932
Missing	1008	577	546	475	285
Caregiver Education					
Up to high school diploma, GED	2022	1147	1084	893	523
Some college, associate's degree	3464	2233	2125	1868	1205
Bachelor's degree	3317	2204	2125	1889	1313
Graduate degree	2981	1863	1771	1591	1089
Missing	17	10	10	9	1
MRI Scanner Manufacturer					
Siemens	7303	4539	4457	4117	2748
GE Medical Systems	2977	2013	1810	1444	1041
Philips Medical Systems	1521	905	848	689	342

Note. Percentages in the *N* row reflect percent of the entire ABCD Study sample (i.e., 11,801), while percentages in the *Sex* row reflect the percent of participants in that column's sample that were assigned female at birth. *MRI 2-Year Follow-Up* indicates the individuals with MRI data collected at baselines (ages 9-10 years) and 2-year follow-up (ages 11-12) visits. The "Other" Race/Ethnicity category includes participants whose caregiver identified them as American Indian/Native American, Alaska Native, Native Hawaiian, Guamanian, Samoan, Other Pacific Islander, Asian Indian, Chinese, Filipino, Japanese, Korean, Vietnamese, Other Asian, Other Race, or as belonging to more than one race. Abbreviations: *QC*: quality control; *sMRI*: structural MRI (i.e., T1-weighted); *dMRI*: diffusion-weighted MRI; *rs-fMRI*: resting-state functional MRI.

© 2024 Bottenhorn KL et al. *JAMA Network Open.*

eReferences

1. Garavan H, Bartsch H, Conway K, et al. Recruiting the ABCD sample: Design considerations and procedures. *Dev Cogn Neurosci*. 2018;32:16-22. doi:10.1016/j.dcn.2018.04.004
2. Volkow ND, Koob GF, Croyle RT, et al. The conception of the ABCD study: From substance use to a broad NIH collaboration. *Dev Cogn Neurosci*. 2018;32:4-7. doi:10.1016/j.dcn.2017.10.002
3. Birn RM, Molloy EK, Patriat R, et al. The effect of scan length on the reliability of resting-state fMRI connectivity estimates. *NeuroImage*. 2013;83:550-558. doi:10.1016/j.neuroimage.2013.05.099
4. Noble S, Spann MN, Tokoglu F, Shen X, Constable RT, Scheinost D. Influences on the Test–Retest Reliability of Functional Connectivity MRI and its Relationship with Behavioral Utility. *Cereb Cortex*. 2017;27(11):5415-5429. doi:10.1093/cercor/bhx230
5. Hamilton CM, Strader LC, Pratt JG, et al. The PhenX Toolkit: get the most from your measures. *Am J Epidemiol*. 2011;174(3):253-260. doi:10.1093/aje/kwr193
6. Casey BJ, Cannonier T, Conley MI, et al. The Adolescent Brain Cognitive Development (ABCD) study: Imaging acquisition across 21 sites. *Dev Cogn Neurosci*. 2018;32:43-54. doi:10.1016/J.DCN.2018.03.001
7. Hagler DJ, Hatton S, Cornejo MD, et al. Image processing and analysis methods for the Adolescent Brain Cognitive Development Study. *NeuroImage*. 2019;202:116091. doi:10.1016/j.neuroimage.2019.116091
8. Fligner MA, Killeen TJ. Distribution-Free Two-Sample Tests for Scale. *J Am Stat Assoc*. 1976;71(353):210-213. doi:10.1080/01621459.1976.10481517
9. Li J, Ji L. Adjusting multiple testing in multilocus analyses using the eigenvalues of a correlation matrix. *Heredity*. 2005;95(3):221-227. doi:10.1038/sj.hdy.6800717
10. Šidák Z. Rectangular Confidence Regions for the Means of Multivariate Normal Distributions. *J Am Stat Assoc*. 1967;62(318):626-633. doi:10.1080/01621459.1967.10482935
11. Desikan RS, Ségonne F, Fischl B, et al. An automated labeling system for subdividing the human cerebral cortex on MRI scans into gyral based regions of interest. *NeuroImage*. 2006;31(3):968-980. doi:10.1016/j.neuroimage.2006.01.021

© 2024 Bottenhorn KL et al. *JAMA Network Open.*
