# Supplementary material for: Adolescent Neurodevelopmental Variance Across Social Strata
Source: JAMA Netw Open. 2024 May 8;7(5):e2410441. doi: 10.1001/jamanetworkopen.2024.10441 (PMC11079691; doi:10.1001/jamanetworkopen.2024.10441)
Supplement: Supplement 2. — Data Sharing Statement [file jamanetwopen-e2410441-s002.pdf]

Data Sharing Statement

Bottenhorn. Adolescent Neurodevelopmental Variance Across Social Strata. *JAMA Netw Open*. Published May 08, 2024. doi:10.1001/jamanetworkopen.2024.10441

Data
Data available: No

Additional Information
Explanation for why data not available: Data analyzed in this manuscript are 4.0 annual curated release (https://dx.doi.org/10.15154/1523041) from the Adolescent Brain Cognitive Development (ABCD) Study and are available through the NIH Data Archive (NDA): nda.nih.gov/abcd.
